# Identification of Key Biomarkers in Systemic Lupus Erythematosus by a Multi-Cohort Analysis

**DOI:** 10.3389/fimmu.2022.928623

**Published:** 2022-07-04

**Authors:** Meilin Wei, Qiguan Dong, Shaoqiu Chen, Junlong Wang, Hua Yang, Qin Xiong

**Affiliations:** ^1^ Department of Endocrinology and Metabolism, The Second Affiliated Hospital of Nanchang University, Jiangxi, China; ^2^ Department of Oncology, General Hospital of Fushun Mining Bureau of Liaoning Health Industry Group, Fushun, China; ^3^ Molecular Biosciences and Bioengineering Program, College of Tropical Agriculture and Human Resources, University of Hawaii at Manoa, Honolulu, HI, United States; ^4^ Chaminade University of Honolulu, Honolulu, HI, United States; ^5^ Department of Endocrinology and Metabolism, The First Affiliated Hospital of Nanchang University, Jiangxi, China

**Keywords:** SLE, transcriptome, diagnosis, immune cells, multi-cohort

## Abstract

Systemic lupus erythematosus (SLE) is an autoimmune disease that affects multiple body systems with heterogeneous clinical manifestations. Since gene expression analyses have been accomplished on diverse types of samples to specify SLE-related genes, single-cohort transcriptomics have not produced reliable results. Using an integrated multi-cohort analysis framework, we analyzed whole blood cells from SLE patients from three transcriptomics cohorts (n=1222) and identified a five-gene signature that distinguished SLE patients from controls. We validated the diagnostic performance of this five-gene signature in six independent validation cohorts (n= 469), with an area under the receiver operating characteristic curve of 0.88 [95% CI 0.7 − 0.96]. This five-gene signature may be associated with the proportion of SLE immune cells, and generalizable across ages and sample types with real diagnostic value for clinical application.

## Introduction

Systemic lupus erythematosus (SLE) is a classic systemic autoimmune disease characterized by aberrant activity of the immune system and formation of nuclear autoantigens and immune complexes resulting in inflammation of multiple organs (1).

The incidence and prevalence of SLE varies among different ethnicities and minority groups and global prevalence is about 0-241/100000 (2). The Lupus nephritis is the most common target-organ manifestation in about 50% patients with SLE and is the most common cause of morbidity and mortality (3). Moreover, other organs involvement such as CNS involvement, cardiovascular disease, lung and skin damage contribute to mortality of SLE.

Without timely diagnosis and treatment or poor control, it will lead to irreversible damage to organs, even cause death (4). With more autoantibody tests available over the past decades, more than 180 autoantibodies can be detected in patients with SLE (5). A systematic literature search and meta-regression of diagnostic data found that the vast majority of SLE patients (97.8% [95% CI 96.8, 98.5]) have positive anti-nuclear antibodies (ANAs), suggesting ANAs are closely associated with SLE (6). Therefore, ANAs are important for diagnosis of SLE and are used as a screening parameter in routine therapy. In recent years, many novel diagnostic markers for SLE have been reported, such as serum Galectin-9, exosomal miRNAs and non-coding RNAs, further studies are needed to confirm their value in diagnosis of SLE (7, 8). There are no universally accepted diagnostic criteria for SLE because of genetic heterogeneity. However, optimized classification criteria for SLE have been widely used based on clinical manifestations and autoimmune serology.

An interplay between genetic predisposition, epigenetic and environmental factors is involved in SLE development and activity. A fraction of genetic loci with SLE susceptibility has been demonstrated (9). The diagnostic accuracy and therapy for SLE have been dramatically improved over time. However, SLE is still a disease with poor prognosis and cause serious harm to the health of patients. Therefore, diagnosing, treating, and identifying novel therapies for SLE is still challenging and more accurate diagnostic markers are needed to assess the SLE. In essence, SLE is an autoimmune disease and we speculated dysregulated gene expression from whole blood or immune cells may provide new insights in SLE diagnosis.

In the study, we analyzed whole blood or immune cells from SLE patients from transcriptomics cohorts and identified novel biomarkers for SLE diagnosis. Additional biomarkers revealed by new technology may help us improve understanding of pathogenesis of SLE.

## Methods

### Study Design and Sample Collection

We conducted a systematic search for expression profiling by array and whole-genome expression datasets that diagnosed SLE patients. We identified 9 datasets and divided them into 3 discovery cohorts (GSE65391, GSE49454, GSE50635) and 6 validation cohorts (GSE10325, GSE30153, GSE37356, GSE27427, GSE39088, GSE11909) from Gene Expression Omnibus (GEO) repository (**Table 1**).

**Table 1:** Demographic of the study population in discovery and validation dataset.

Dataset	Sample type	Sample size	Annotation
GSE49454	Whole Blood	177	Discovery
GSE10325	CD4 T cells, CD19 B cells	67	Validation
GSE50635	Whole Blood	49	Discovery
GSE65391	Whole Blood	996	Discovery
GSE30153	B cells	26	Validation
GSE37356	Monocytes	72	Validation
GSE27427	Neutrophils	47	Validation
GSE39088	Whole Blood	142	Validation
GSE11909	Whole Blood	115	Validation

In discovery cohorts, three microarray was performed to identify differentially expressed genes in SLE and control group (Control VS SLE). Expression profiling of whole blood samples from two validation cohorts (GSE39088, GSE11909). Additional four validation cohorts (GSE10325, GSE30153, GSE37356, GSE27427), patients samples obtained from CD4 T cells/CD19 B cells, B cells, Monocytes and Neutrophils, respectively.

### Statistics and Bioinformatics Analyses

Affymetrix chips were normalized using GC Robust Multi-Array Average (GCRMA) and all other chips were normalized using normal-exponential correction and quantile normalization. Log2 normalization was performed in all arrays before analysis. DerSimonian-Laird random effects were used to combine gene expression effect sizes *via* Hedges’g (10). For differential expression, we set significance thresholds of FDR less than 1% and an effect size greater than 0.8 fold (in non-log space). All analyses were executed with R software (version 4.1.3).

### Establishment of SLE Score

Using the MetaIntegrator R package, we ran a forward search to identify the most miserly gene signature that maximizes diagnostic performance (11). Each sample’s SLE score is derived by subtracting the mean expression of the downregulated genes from the mean expression of the upregulated genes.

### Deconvolution Analysis

Through the ImmuneDeconv package, we conducted cell-mixture deconvolution analysis (12). Briefly, each microarray dataset was converted into a gene-expression matrix. The methodology uses a list of genes, which are Characteristics for each cell type, as markers. Based on the expression values of signature genes, each cell type is quantified separately.

## Results

### The Five-Gene Signature Diagnostic of SLE in Three Discovery Cohorts

**Figure 1:**
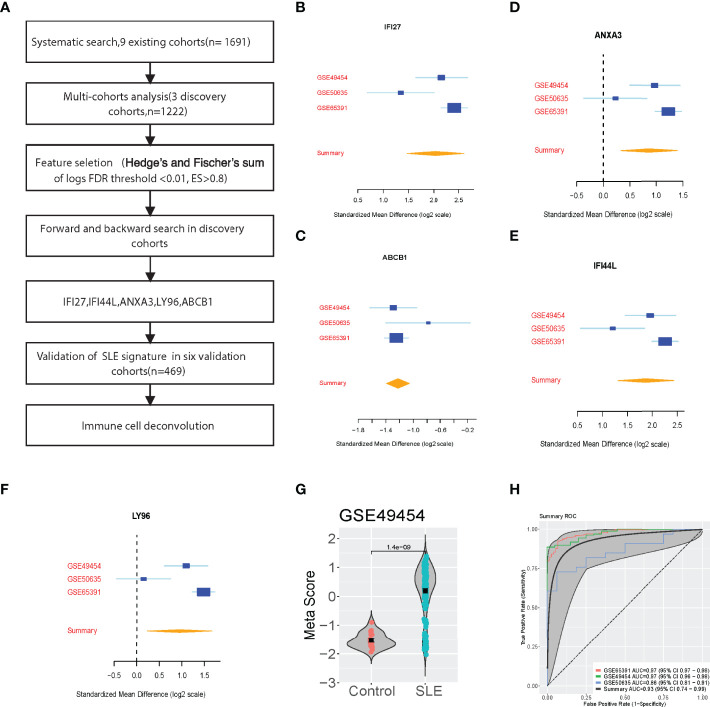
Discovery of the five-Gene signature diagnosis of SLE. **(A)** Multi-cohort analysis workflow for identifying and validating the 5-gene signature. **(B–F)** 5-gene signature from the forward search. **(G)** A representative violin plot illustrating the performance of the 5-gene signature to differentiate SLE from Control in one of the discovery cohorts (GSE49454, **Supplementary Figure S1**). **(H)** ROC curves of patients with SLE versus controls in three discovery datasets.

We conducted a systematic search for data on whole-genome expression in whole blood from patients with SLE. Our multicohort analysis found 93 significantly differentially expressed genes (FDR < 0.01, effect size > 0.8-fold) between patients who with SLE vs Control in the three discovery cohorts (N = 1222). Using forward search, we specified a signature of five significantly differentially expressed genes in SLE and Control that was optimized for diagnostic performance (**Figure 1A**). The forest plots showed that the 5 selected genes were differentially expressed (4 up-regulated, 1 down-regulated) according to the forward search (**Figures 1B–F**). In each sample, we calculated a SLE score by subtracting the mean expression of the one down-regulated gene from the mean expression of the up-regulated genes (**Figure 1G** and **Figure S1**). There was a significant difference between patients with SLE and Control in the discovery cohorts with a summary area under the curve (AUC) equal to 0.93 (95% confidence interval 0.74-0.99, **Figure 1H**).

### Validation of the Five-Gene Signature in Six Independent Validation Cohorts of SLE Patients

**Figure 2:**
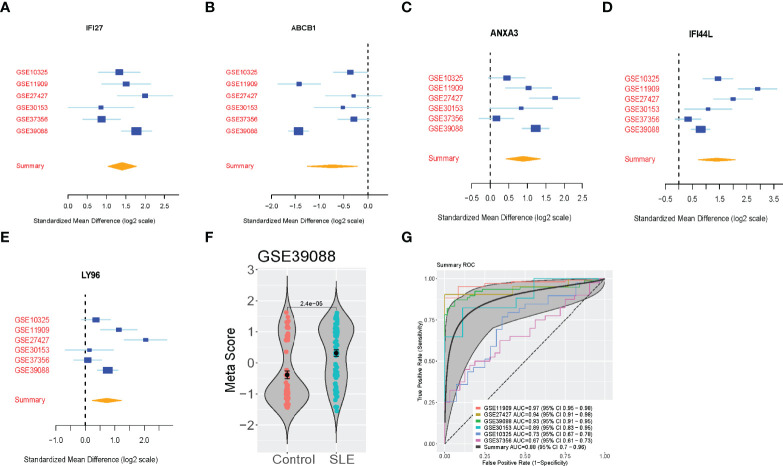
Validation of the 5-Gene signature diagnosis of SLE. **(A–E)** The up-regulated genes and down-regulated genes in independent validation cohorts. **(F)** A representative violin plot illustrating the performance of the five-gene signature to differentiate SLE from Control in one of the discovery cohorts (GSE39088). The Wilcoxon p value and error bars are shown. **(G)** ROC curves of patients with SLE versus controls in six independent validation cohorts.

We then validated the five-gene set in the six validation cohorts (N=469). We validated the up-regulated and down-regulated genes (**Figures 2A–E**). Also, we calculated the SLE score of each sample in the validation cohorts and meta score of each dataset (**Figure 2F** and **Figure S1**). Although the considerable heterogeneity between datasets, including sample type, race, platform, the SLE scores accurately identified SLE patients in all six datasets (summary AUC=0.88 [95% CI 0.7-0.96]) (**Figure 2G**). Notably, this signature could effectively discriminate the SLE patients for the four-validation dataset (GSE11909, GSE27427, GSE39088, GSE30153). However, the AUC of SLE scores in the GSE37356 (Monocytes) and GSE10325 (CD4 T cells, CD19 B cells) dataset were 0.67 and 0.73, respectively.

### Deconvolution of Immune Cell Types

**Figure 3:**
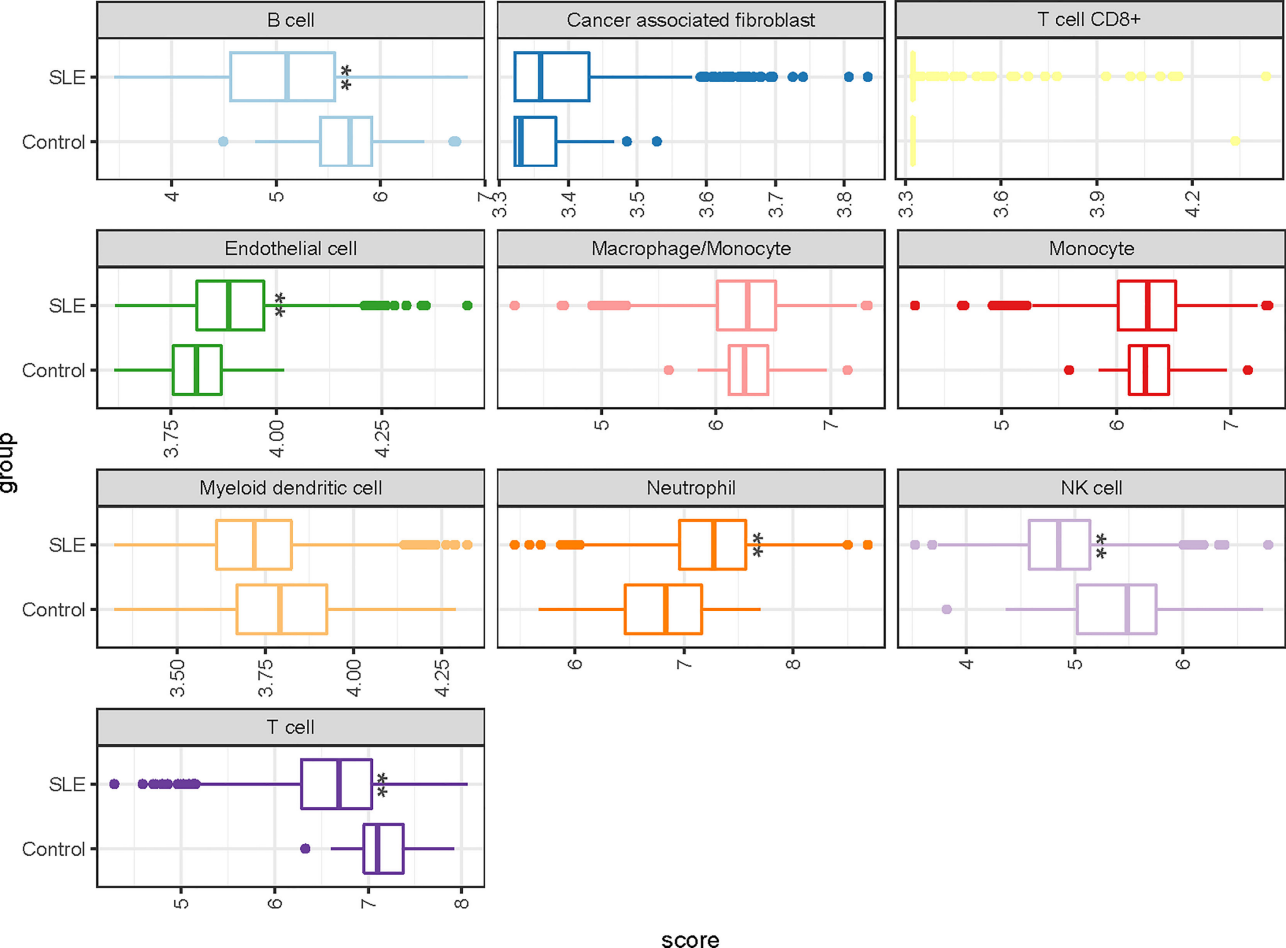
A representative boxplot illustrating the analysis of the enrichment profiles of immune cells in deconvolution of cell mixtures (GSE65391). * P-value < 0.05; ** P-value < 0.01.

We also examined whether the five-gene signature is enriched in some immune cell types by using ImmuneDeconv analysis, which is based on publicly available whole-genome expression profiles from 10 molecular immune cells. We found that the percentages of NK Cell, B cells and neutrophils were significantly changed in the SLE group. Interestingly, no statistically consistent and reproducible differences in the proportions of T cells and monocytes (**Figure 3** and **Figure S2**).

## Discussion

SLE is primarily caused by autoantibodies-induced tissue injury. An irregular immune response and inflammation are also significant pathological processes in SLE. Although numerous studies have been conducted, early diagnosis of SLE remains a challenging diagnostic problem, and many studies have suffered from a reproducibility crisis. The reproducibility crisis has been exacerbated by the fact that results from homogeneous, single-center studies do not generally generalize to heterogeneous populations in the real world. By combining data from various populations into one study, multi-cohort gene expression analysis has improved reproducibility. Here, through the use of a large, multicenter dataset of various types of samples, we identified five diagnostic biomarkers for patients with SLE (IFI27, IFI44L, ANXA3, LY96, ABCB1). Furthermore, we utilized six independent validation sets to verify the diagnostic power of the five-gene signature.

There is strong evidence from previous studies that four of the above five genes are involved in SLE pathogenesis and therapeutics. In patients with SLE, the expression of IFI27, IFI44L, and ANXA3 were up-regulated as compared to controls (13, 14). A previous study demonstrated that IFI44L promoter methylation as a blood biomarker for systemic lupus erythematosus (15). Moreover, IFI27, IFI44L, and ANXA3 are associated with highly expressed interferon-stimulated genes. Up-regulation of IFI27 (the interferon alpha-inducible protein27) expression may be associated with inflammatory response (16), and is involved in the progression of an HIV infection by regulating immune response (17). P-glycoprotein encoded by ABCB1 gene is an ATP-dependent drug efflux pump, which has been detected in some autoimmune disease, such as SLE (18). And ABCB1 gene polymorphisms may be associated with clinical features of SLE (19). Additionally, synovial tissue from SLE patients up-regulated IFI27 and IFI44L (20). Notably, we first demonstrate that LY96 genes are novel markers of SLE in this study. Molecular analysis suggests that the LY96 coding protein binds to toll-like receptor 4 on the cell surface and grants responsiveness to lipopolysaccharide (LPS), thus connecting the receptor and LPS signaling (21). The effectiveness of LY96 to another type of auto-immune disease, namely Rheumatic, has also been reported (22).

Cell-type enrichment analysis suggest that reduced abundances of NK Cell, and B cells in patients with SLE. Interestingly, there was no significant difference in monocyte proportions between SLE and the control group, and diagnostic power was also limited in the independent validation set (AUC=0.67, [95% CI 0.61-0.73]).

In summary, we identified five genes that are significantly associated with SLE diagnosis. In addition, this result was validated across nine datasets and multiple types of human blood-born samples. This suggests that these genes could serve as biomarkers for the diagnosis of SLE in real clinical application. Understanding the mechanism of SLE pathogenesis through the five-gene signature can be beneficial. In a follow-up study, we hope to gain a deeper understanding of their possible functions.

## Data Availability Statement

The datasets presented in this study can be found in online repositories. The names of the repository/repositories and accession number(s) can be found in the article/**Supplementary Material**.

## Ethics Statement

The studies involving human participants were reviewed and approved by Baylor Institute for Immunology Research, UT Southwestern Medical Center, and Mayo Clinic. The patients/participants provided their written informed consent to participate in this study.

## Author Contributions

MW, QX, and SC established the concept and the investigation. MW, QD, SC, JW collected and processed data. MW, SC drafted the manuscript, and all authors reviewed the manuscript and approved the version for publication. All authors contributed to the article and approved the submitted version.

## Conflict of Interest

The authors declare that the research was conducted in the absence of any commercial or financial relationships that could be construed as a potential conflict of interest.

## Publisher’s Note

All claims expressed in this article are solely those of the authors and do not necessarily represent those of their affiliated organizations, or those of the publisher, the editors and the reviewers. Any product that may be evaluated in this article, or claim that may be made by its manufacturer, is not guaranteed or endorsed by the publisher.
