# Supplementary material for: Identification of Key Biomarkers in Systemic Lupus Erythematosus by a Multi-Cohort Analysis
Source: Front Immunol. 2022 Jul 4;13:928623. doi: 10.3389/fimmu.2022.928623 (PMC9289109; doi:10.3389/fimmu.2022.928623)
Supplement: Supplementary file 1 [file Presentation_1.pdf]

Identification of key biomarkers in systemic lupus erythematosus by a multi-cohort analysis

Figure S1 Violin plots illustrating the Meta score of the 5-gene set to separate SLE from control. Discovery(Left) and Validation(Right)

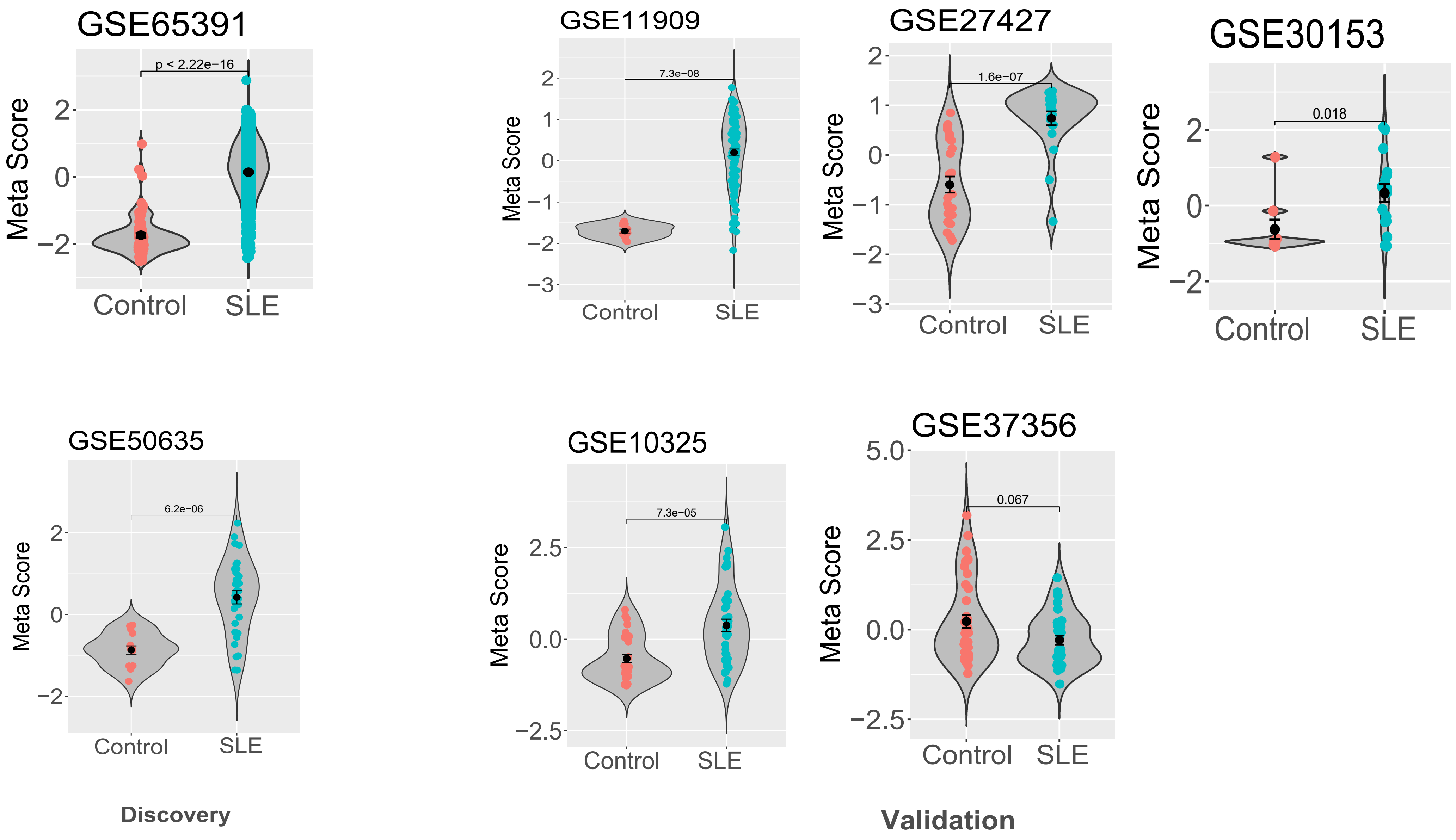

Figure S2 The analysis of the enrichment profiles of the five differentially expressed genes in deconvolution of cell mixtures (Validation: GSE11909, GSE39088, GSE49454, GSE50635) . *,p-vlaue < 0.05; **,p-vlaue < 0.05

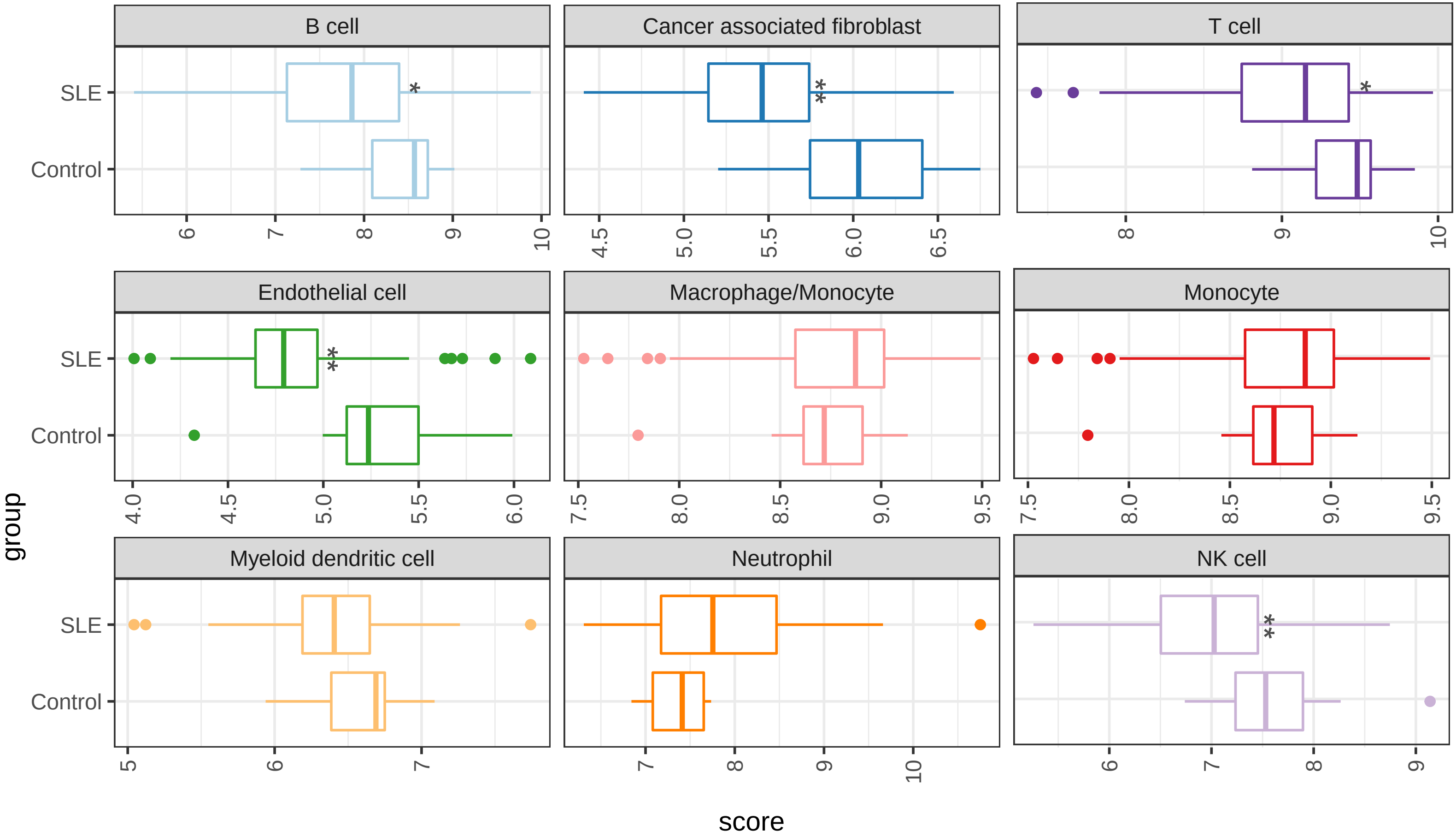

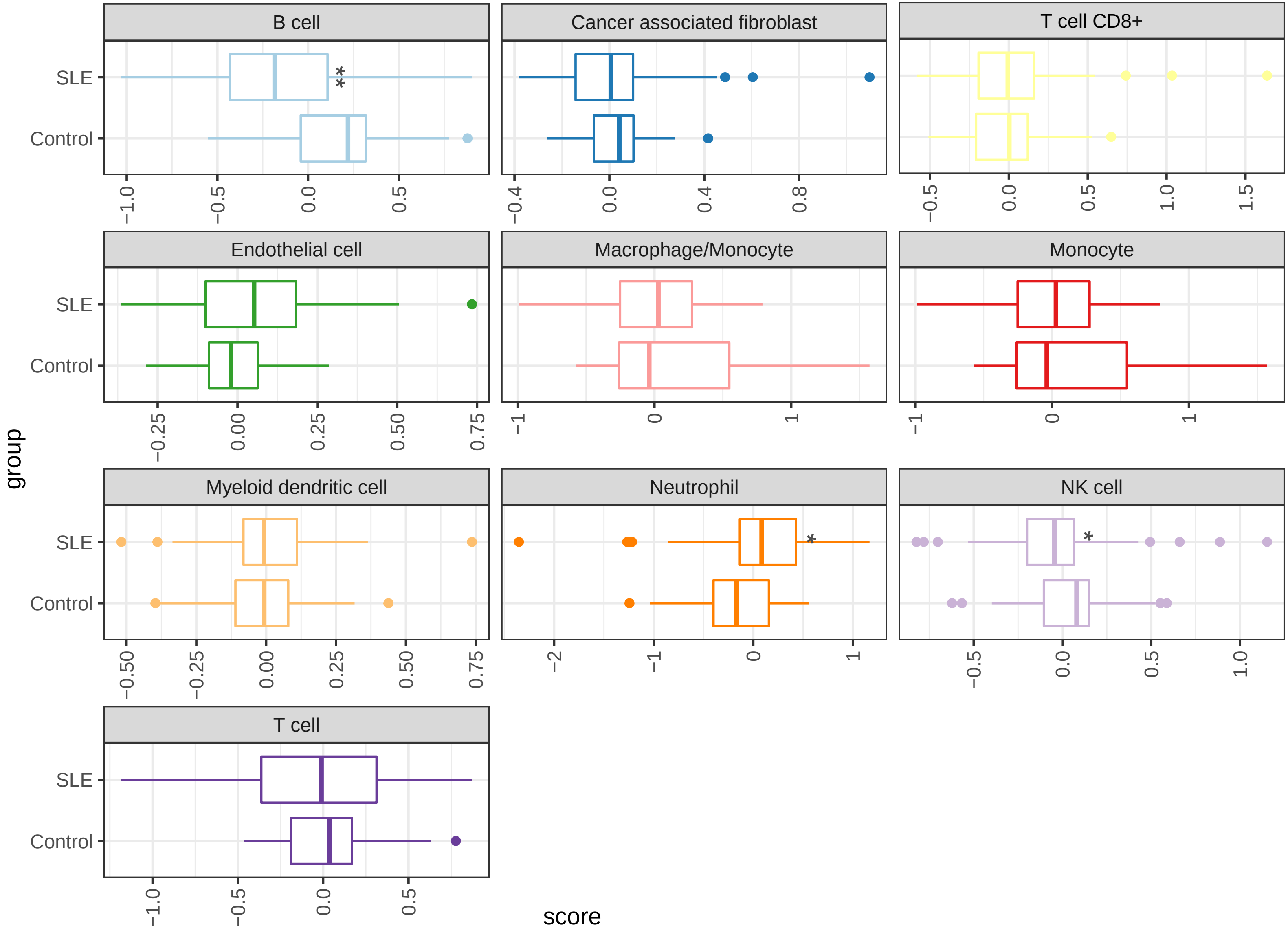
B cell
Cancer associated fibroblast
T cell CD8+
Endothelial cell
Macrophage/Monocyte
Monocyte
Myeloid dendritic cell
Neutrophil
NK cell
T cell
SLE
Control
group
score
**
*
*

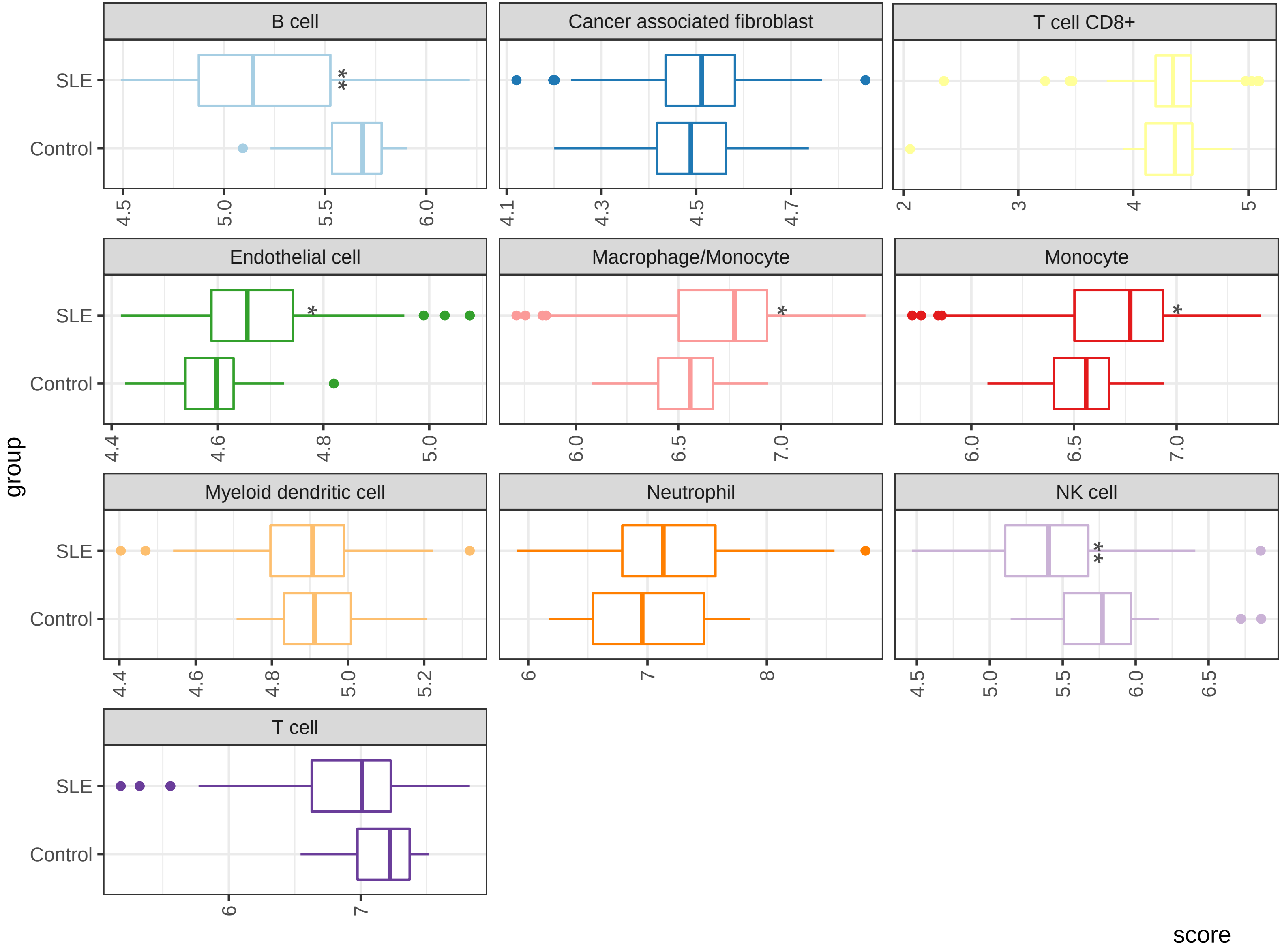
B cell
Cancer associated fibroblast
T cell CD8+
Endothelial cell
Macrophage/Monocyte
Monocyte
Myeloid dendritic cell
Neutrophil
NK cell
T cell
SLE
Control
group
score
**
*
4.5
5.0
5.5
6.0
4.1
4.3
4.5
4.7
2
3
4
5
4.4
4.6
4.8
5.0
6.0
6.5
7.0
5.2
6
7
8

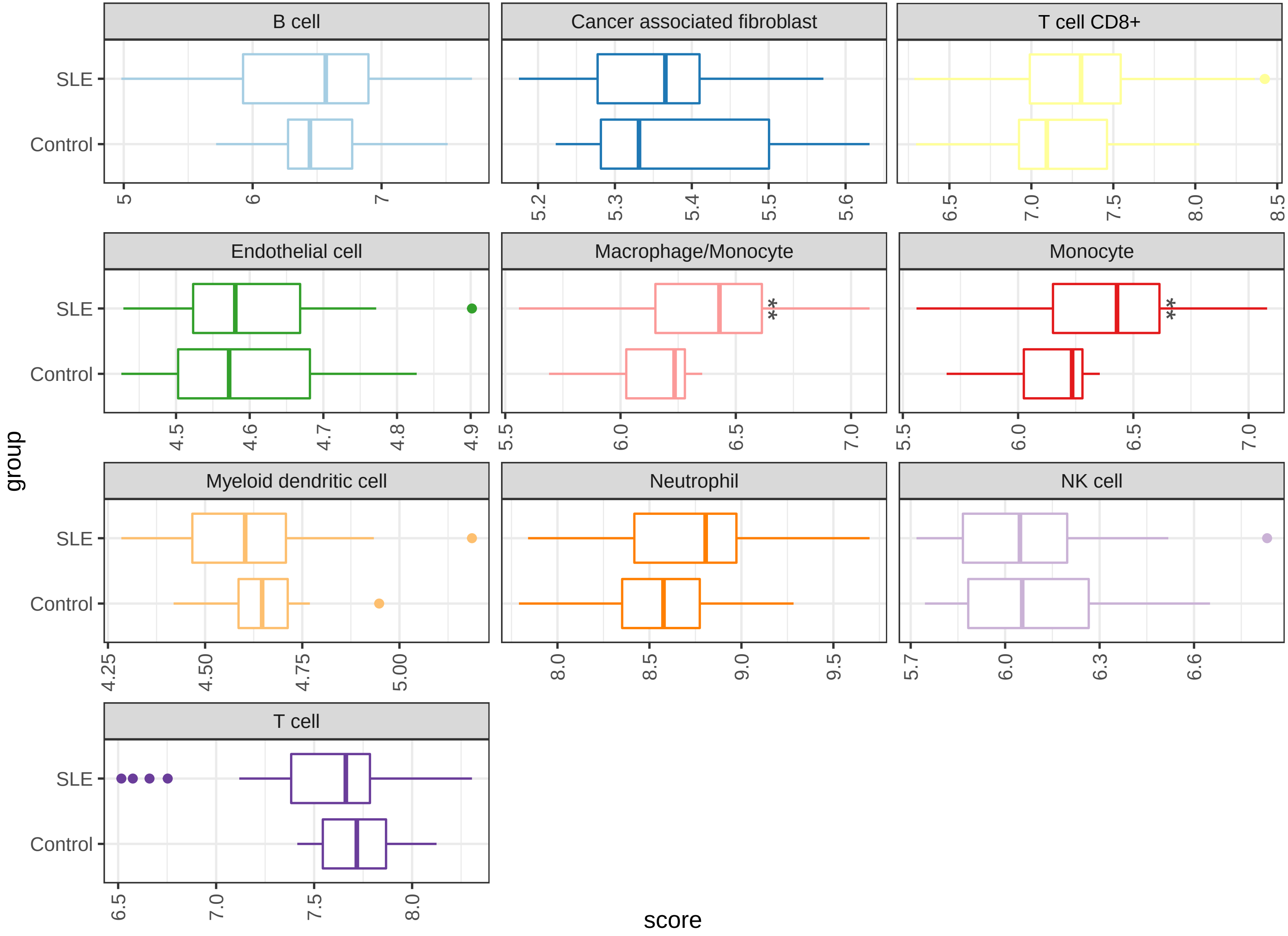

B cell
Cancer associated fibroblast
T cell CD8+
Endothelial cell
Macrophage/Monocyte
Monocyte
Myeloid dendritic cell
Neutrophil
NK cell
T cell
SLE
Control
**
group
score
